# Linguatuliasis in Germany

**DOI:** 10.3201/eid1206.051413

**Published:** 2006-06

**Authors:** Dennis Tappe, Ralf Winzer, Dietrich W. Büttner, Philipp Ströbel, August Stich, Hartwig Klinker, Matthias Frosch

**Affiliations:** * University of Würzburg, Würzburg, Germany;; † Bernhard-Nocht-Institute for Tropical Medicine, Hamburg, Germany;; ‡ Medical Mission Hospital, Würzburg, Germany

**Keywords:** Linquatula serrata, pulmonary infection, pentastomiasis, Germany, letter

**To the Editor:** Pentastomids or tongue worms are a unique group of vermiform parasites, phylogenetically related to arthropods (*1*). Of the many pentastomid species, only a few, including *Linguatula serrata*, infect humans. The adult parasites are long, flat, or annulated and have 4 hooks surrounding a central mouth. Adult *L. serrata* inhabit the nasal passages and paranasal sinuses of wild and domestic canids, which serve as definitive hosts. Infective eggs containing larvae are discharged into the environment by nasopharyngeal secretions and are ingested by herbivores, the natural intermediate hosts. Humans can become dead-end intermediate hosts; visceral linguatuliasis then develops (*2*) if infective eggs are ingested. The liver is the organ most often involved (*3**–**5*), but the lung (*4**,**6**,**7*) or other organs (*4**,**8*) may be affected. Parasites may also be found in lymph nodes. In the viscera, the primary 4-legged larva molts several times and eventually forms the legless nymph. Lesions due to *Linguatula* may be confused with malignancy, particularly in the lung (*6*).

We describe a recent infection with *L. serrata* in Germany in a patient who had pulmonary symptoms and in whom malignancy was suspected. The patient was a 39-year-old man of Russian origin who had been living in Germany since 1999. He was admitted to the hospital with weight loss, night sweats, chest pain, and coughing. He had been a smoker for 20 years, and his past medical history included pneumonia and sinusitis in 1989 during his military service at Lake Baikal, Russia. The patient had been living in a farmhouse in Karaganda, Kazakhstan, until he immigrated to Germany.

A chest radiograph and computed tomographic scan showed multiple, small lesions in both lungs. Malignancy was suspected, and a bronchoscopy was performed. Numerous granulomatous nodules were discovered. Thoracotomy was performed, and stringlike nodules on the pleural surface were resected. Except for a mild eosinophilia (7%, 500 cells/μL), the leukocyte count was normal. All other parameters, including C-reactive protein levels, angiotensin-converting enzyme, and tumor markers were normal. Histologic examination of the nodules showed a targetoid appearance with a sharp demarcation from the surrounding lung tissue by a thick fibrocollagenous capsule. In the center of the nodules, a transverse section (Figure, right inset) and a longitudinal section (Figure, main panel) of a parasite were visible. The parasite had a chitinous cuticle ≈2.5 μm thick and cuticular spines 20–30 μm long. The spines and the serrated aspect are characteristic for *L. serrata*, a pentastome. Ringlike structures in the body wall were interpreted as sclerotized openings, a key feature of pentastomes. In close contact to host tissue, a shed cuticle was visible and assigned to the previous instar larva. The biometric data of the parasite were comparable to those measured by others (6,9). Hooks, typical for the oral armature of pentastomes, were found by serial sectioning (Figure, left inset). Except for some subcuticular glands, the parasite's inner organs were no longer distinguishable. The patient was initially treated with albendazole before the histologic diagnosis of linguatuliasis was established. Findings from magnetic resonance imaging of the abdomen were unremarkable, and no further lesions appeared during 12 months of followup. Intermittent cough and chest pain remained, possibly due to scar tissue and the remains of the nymphs.

**Figure:**
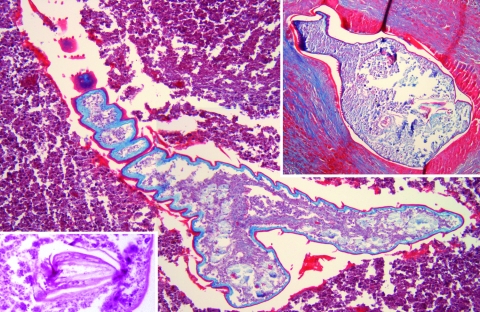
*Linguatula serrata* nymphs in lung tissue. Main panel shows the parasite's serrated nature and the cuticular spines (magnification ×200, Masson trichrome stain). Right upper inset, pulmonary nodule with prominent fibrotic reaction and shed cuticle around 1 nymph (magnification ×200, Masson trichrome stain). Left lower inset, detailed view of 1 parasite hook (magnification ×630, hematoxylin and eosin stain).

At the beginning of the last century, visceral linguatuliasis of humans occurred frequently in Germany. In 1904 and 1905, among 400 autopsies in Berlin, 47 (11.8%) remains were infected with *L. serrata* (*7*). In contrast, reports of human infections are now rare. Our report is the first recent case description in Germany. Where the patient acquired the infection is unknown. *L. serrata* has a worldwide distribution. Recent cases have been reported from China (*4*) and Italy (*6*). An increasing number of infections can be suspected in the Western Hemisphere because of incremental travel to linguatuliasis-endemic areas. Humans are usually tolerant to nymphal pentastomid infections, and most patients are asymptomatic (*4*). The living nymph provokes little inflammation, whereas the death of the parasite leads to a prominent host response (*2*). Most findings of visceral linguatuliasis are made at autopsy (*4**,**6*), and the parasites are mainly located in the liver (*3**–**5*). Infection of the lung is rare (*6**,**7*). The nymphs in human granulomas are typically degenerated at the time of examination (*3**,**6**,**9*), but the cuticle with its associated structures remains visible for some time (*2*). Histopathologic diagnosis is guided by the presence of remnants of the cuticle with sclerotized openings and by calcified hooks. Among pentastomids observed in humans, only *L. serrata* has prominent spines (*2**–**4*). In contrast to trematodes, the spines protrude from the cuticle and do not end in the body wall of the parasite. Diagnosis should be made etiopathologically, subetiopathologically, or presumptively on the basis of whether entire nymphs, cuticle-associated structures, or pearly lesions ("*Linguatula* nodules" [10]) with targetoid appearance are found (*4*). The differential diagnosis includes malignancies and tuberculosis because of the radiologic coinlike appearance. On histologic examination, one must distinguish between tissue-inhabiting diptera larvae, infections with metacestodes, trematodes, tissue filariids, and gnathostomiasis. Once diagnosis is established, no treatment is necessary (*3*) for the parasites will degenerate after some time, and no effective antiparasitic therapy exists. Avoiding contact with canine saliva and drinking water used by dogs or wild canids prevents this infection.
